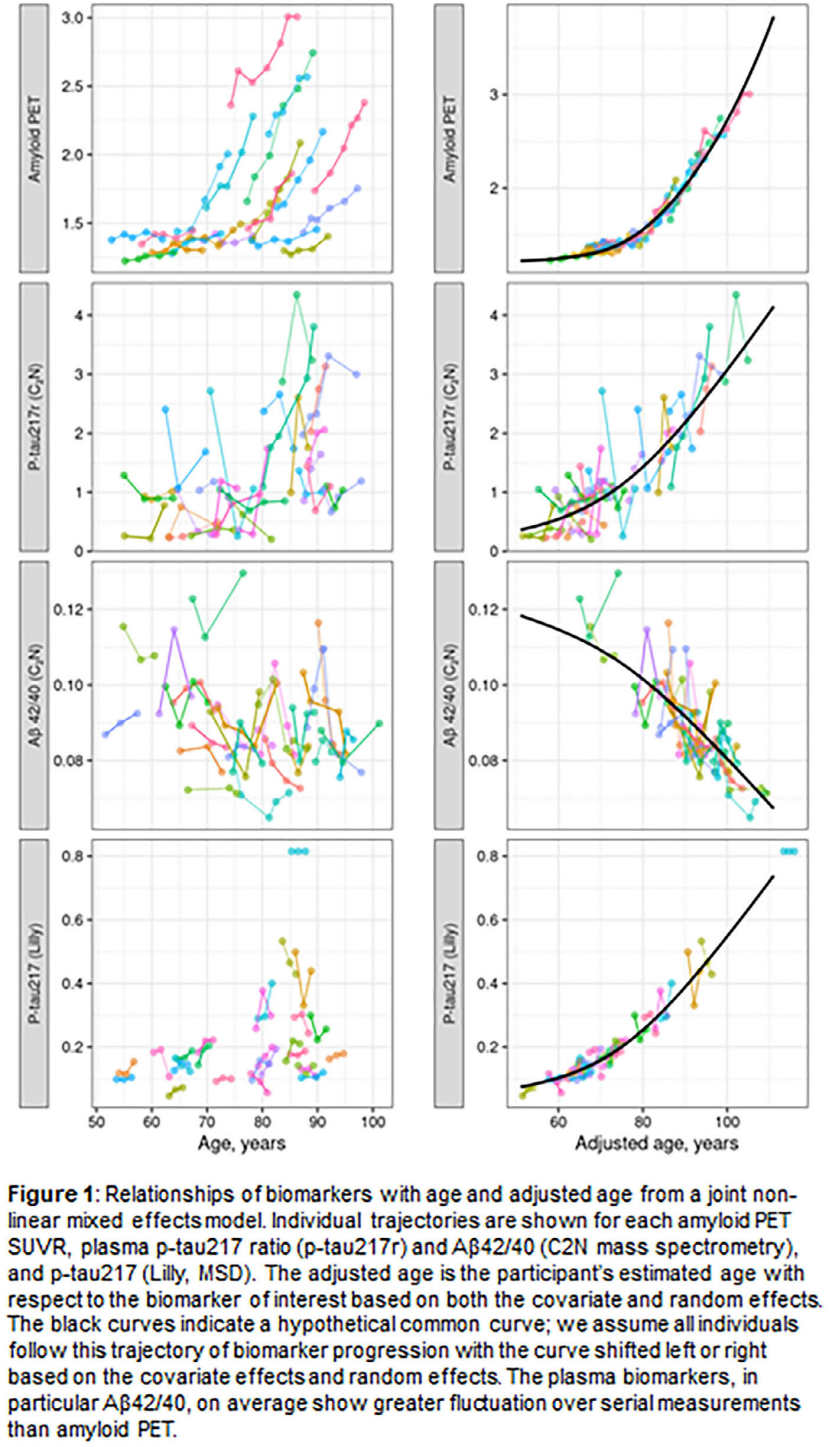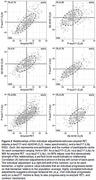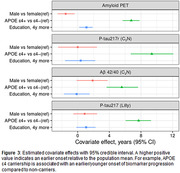# Modeling the temporal evolution of mass spectrometry plasma biomarkers

**DOI:** 10.1002/alz.090361

**Published:** 2025-01-09

**Authors:** Petrice M Cogswell, Emily S. Lundt, Terry M. Therneau, Michael E. Griswold, Jonathan Graff‐Radford, Alicia Algeciras‐Schimnich, Val J. Lowe, Christopher G. Schwarz, Matthew L. Senjem, Jeffrey L. Gunter, David S. Knopman, Prashanthi Vemuri, Ronald C. Petersen, Clifford R. Jack

**Affiliations:** ^1^ Mayo Clinic, Rochester, MN USA; ^2^ University of Mississippi Medical Center, The MIND Center, Jackson, MS USA; ^3^ Department of Radiology, Mayo Clinic, Rochester, MN USA

## Abstract

**Background:**

Plasma Aβ42/40 and p‐tau217 can predict amyloid positivity in cross‐sectional studies. However, it is unclear how plasma biomarkers perform longitudinally, which is important to inform their utility in tracking disease progression. The goal of this study is to describe temporal evolutions of plasma Aβ42/40 and ptau217 ratio (p‐tau217r) measured via mass spectrometry, p‐tau217 measured via an immunoassay, and amyloid PET.

**Methods:**

We included participants in the Mayo Clinic Study of Aging with amyloid PET (PiB) and plasma Aβ42/40 + ptau217r (mass spectrometry, C_2_N) and/or p‐tau217 (Lilly, MSD). We fit a joint non‐linear mixed effects model with Aβ42/40, p‐tau217r, p‐tau217 (Lilly), and amyloid PET as outcomes and age, sex, APOE ε4 carriership and education as covariates. The model included a per‐participant adjustment (time‐shift) for each biomarker indicating how much earlier or later in time the biomarker was estimated to progress relative to the average participant, a correlation (R) between adjustments for each pair of biomarkers, and the impact of covariates on each biomarker’s timing.

**Results:**

The study included 1459 participants, age 77 (10) years, 44% female, 82% cognitively unimpaired. Mean (SD) years of followup was 5.6(3.0) years for mass spectrometry plasma measures, 2.5(0.7) years for p‐tau217 (Lilly), and 4.6(3.3) for amyloid PET. Model fits are shown for each biomarker, with greater variability in serial measurements for plasma than PET (Figure 1). Individual adjustments between each of the plasma biomarkers and amyloid PET were highly correlated: p‐tau217r, R=0.73 (95%CI: 0.67, 0.78); p‐tau217 (Lilly), R=0.66 (0.62, 0.7); Aβ42/40, R=0.6 (0.52, 0.68) (Figure 2). The p‐tau217r and Aβ42/40 individual adjustments were modestly correlated, R=0.39 (0.28, 0.49). APOE ε4 carriership had the strongest covariate effect on all biomarkers, e.g. p‐tau217r progression 9.4 (6.7, 12) years earlier (Figure 3).

**Conclusions:**

Plasma Aβ42/40 and p‐tau217 measured with currently available assays may both be amenable to longitudinal modeling, although the degree of variability in Aβ42/40 over time may pose challenges. Findings suggest that both plasma Aβ42/40 and p‐tau217r may provide information regarding an individual’s timing of progression on amyloid PET; lower correlations with each other than amyloid PET suggests that each analyte may provide complimentary information.